# A challenging coexistence of central diabetes insipidus and cerebral salt wasting syndrome: a case report

**DOI:** 10.1186/s13256-018-1678-z

**Published:** 2018-07-17

**Authors:** Maria Manuel Costa, César Esteves, José Luís Castedo, Josué Pereira, Davide Carvalho

**Affiliations:** 1 Department of Endocrinology, Diabetes and Metabolism of Centro Hospitalar de São João, Alameda Prof. Hernâni Monteiro, 4200-319 Porto, Portugal; 2 Faculty of Medicine, University of Porto, Alameda Prof. Hernâni Monteiro, 4200-319 Porto, Portugal; 3 Instituto de Investigação e Inovação da Saúde da Universidade do Porto, Alameda Prof. Hernâni Monteiro, 4200-319 Porto, Portugal; 4 Department of Neurosurgery of Centro Hospitalar de São João, Alameda Prof. Hernâni Monteiro, 4200-319 Porto, Portugal

**Keywords:** Hyponatremia, Central diabetes insipidus, Cerebral salt wasting syndrome, Pituitary

## Abstract

**Background:**

Combined central diabetes insipidus and cerebral salt wasting syndrome is a rare clinical finding. However, when this happens, mortality is high due to delayed diagnosis and/or inadequate treatment.

**Case presentation:**

A 42-year-old white man was referred to neurosurgery due to a non-functional pituitary macroadenoma. He underwent a partial resection of the tumor on July 2, 2015. On the day following surgery he presented polyuria with sodium 149 mEq/L, plasma osmolality 301 mOsm/kg, and urine osmolality 293 mOsm/kg. He started nasal desmopressin 0.05 mg/day with good response. He was already on dexamethasone 4 mg and levothyroxine 75 mcg due to hypopituitarism after surgery. On July 9 he became confused. Cerebral computed tomography was performed with no significant changes. His natremia dropped to 128 mEq/L with development of polyuria despite maintenance of desmopressin dose. His hemoglobin and hematocrit rose from 9.1 g/L to 11.6 g/L and 27.5 to 32.5, respectively. His thyroid function was normal and he was on hydrocortisone 30 mg/day. At 12 p.m. 150 mg/hydrocortisone infusion was initiated, but sodium did not increase. Plasma and urine osmolality were 264 mOsm/kg and 679 mOsm/kg, respectively. At 4 p.m. hydrocortisone was increased and hypertonic saline replacement started. Two hours later he was dehydrated with polyuria and vomiting, and natremia of 124 mEq/L. Hyponatremia was very resistant to treatment despite hypertonic saline replacement, hence desmopressin was suspended. The following day, urine spot analysis showed that natriuresis was 63 mEq/L with serum sodium 132 mEq/L. This was interpreted as a cerebral salt wasting syndrome and control was achieved with aggressive hypertonic saline replacements and fludrocortisone 0.1 mg/three times a day.

**Conclusions:**

We present a rare case of a patient with diabetes insipidus and cerebral salt wasting syndrome, who was successfully treated. Hyponatremia in a patient with diabetes insipidus may erroneously be interpreted as inadequate diabetes insipidus control or as syndrome of inappropriate antidiuretic hormone secretion, leading to therapeutic errors. Thus, all clinical and analytical data should be evaluated together for early and correct diagnosis.

## Background

Fluid and electrolytes disorders are not rare in patients after brain surgery, especially in surgery that involves the pituitary gland. Hyponatremia can occur in 8 to 35% of patients following pituitary surgery [1, 2].

There are a lot of causes for water and electrolytic disturbances and, among them, transient diabetes insipidus (DI) has been found to be the most common followed by syndrome of inappropriate antidiuretic hormone secretion (SIADH), cerebral salt wasting syndrome (CSWS), and transient hyponatremia [3–5]. DI is defined as the concomitant presence of inappropriate hypotonic polyuria, which is urine output > 3 L/24 hours and urine osmolality (uOsm) < 300 mOsm/kg, in the presence of high or normal serum sodium (Na) due to decreased secretion of antidiuretic hormone (ADH), leading to the inability to concentrate the urine and subsequent excretion of large volumes of dilute urine [6]. Rarely, after surgery, patients may exhibit a pattern known as triphasic DI: an early polyuric phase, within 24 to 48 hours after surgery; an antidiuretic phase due to an uncontrolled release of vasopressin, usually 5 to 8 days after surgery, which can produce hyponatremia and SIADH; and a final polyuric phase which is often permanent [7].

CSWS is defined as renal loss of Na during intracranial disorders leading to hyponatremia and a decrease in extracellular fluid volume. It is a rare and alarming condition, which is predominantly associated with subarachnoid hemorrhage, but can also occur after head injury, neurosurgery, intracranial neoplasm, or cerebral infection [4, 8]. It was first described in 1950 as an explanation for the natriuresis and hyponatremia that sometimes is associated with cerebral disease [9]. The incidence of CSWS is unclear; the condition is characterized by hyponatremia caused by primary renal salt loss and subsequent polyuria, natriuresis, and hypovolemia. The mechanism underlying CSWS is unclear, but may involve increased levels of circulating natriuretic factors, together with decreased sympathetic input to the kidney. These factors increase urinary Na excretion and diminish effective arterial blood volume, which stimulates baroreceptors on ADH release. Unlike SIADH, ADH release in CSWS is an adequate response to volume depletion. Among patients with central nervous system (CNS) disease, CSWS is a much less common cause of hyponatremia than SIADH [3, 5, 10–12].

DI and CSWS rarely occur simultaneously. There are only a few cases described in the literature of this combination, with a high mortality or vegetative state associated with them [8, 13, 14]. This unfavorable prognosis occurs due to frequent misdiagnosis or incorrect Na correction, thus representing a diagnostic and therapeutic challenge.

We present a rare case of a patient with DI and CSWS who was successfully treated, and we also review the literature regarding this combination.

## Case presentation

**Fig. 1:**
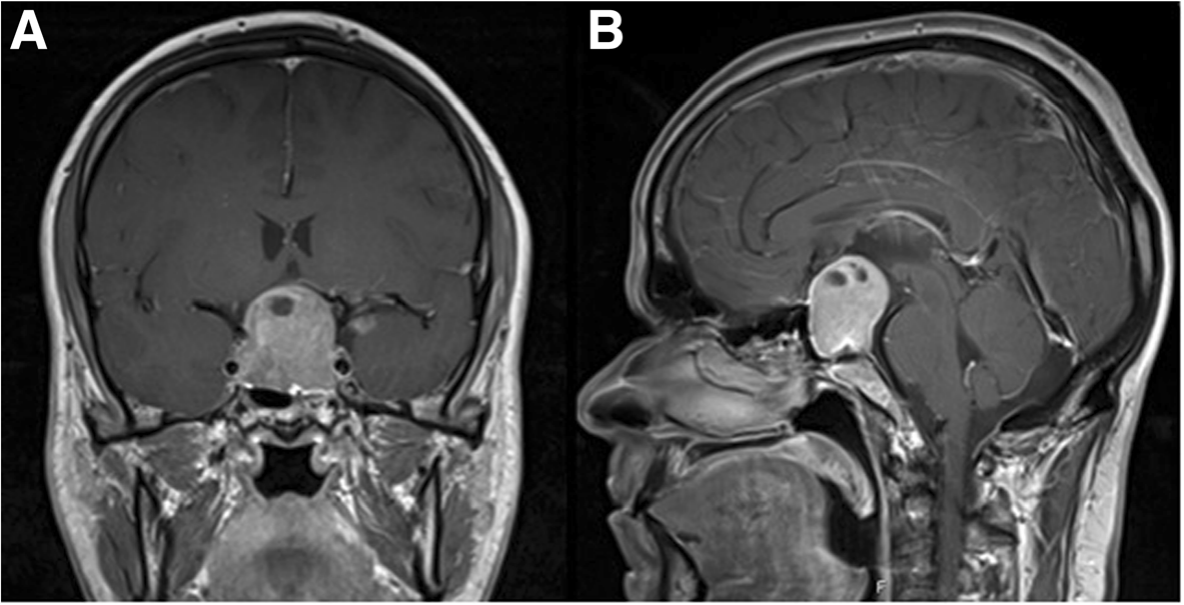
Pituitary magnetic resonance imaging. T1-weighted coronal view (**a**) and sagittal view (**b**) post-gadolinium showing a large macroadenoma

A 42-year-old white man was referred to neurosurgery due to a non-functional pituitary macroadenoma with bitemporal hemianopsia associated. Pituitary magnetic resonance imaging showed a large sellar and suprasellar mass with invasion of cavernous sinuses bilaterally and with superior stretching and bulging of the optic chiasm (Fig. 1). His past medical history included depression, but he was not medicated for this.

He was admitted to our neurosurgery department and underwent partial resection of the tumor by subfrontal approach on July 2, 2015. The tumor was large, but the surgery was no more invasive than the usual pituitary surgery, and there was no section of the pituitary gland.

On the first postoperative day, he presented polyuria of 200 mL/hour with Na 149 mEq/L, plasma osmolality (pOsm) 301 mOsm/kg, uOsm 293 mOsm/kg, and complained of being thirsty. He was receiving an intravenous infusion of 150 mg of hydrocortisone, dexamethasone 4 mg every 8 hours over 24 hours, 1500 ml of intravenously administered isotonic saline, and free water ingestion (Table 1). His plasma glucose levels were between 113 and 138 mg/dL, and his spot analysis did not show glycosuria. His condition was interpreted as DI, and he started nasal desmopressin 0.05 mg/day with good response. On July 5 his Na was 142 mEq/L with resolved polyuria.

**Table 1:** Data of the patient regarding diabetes insipidus and cerebral salt wasting syndrome

Postoperative day	0	1	3	6	7	8	9	11	12
	Day of surgery	DI			CSWS				
Na plasma (mEq/L)	145	149	142	137	128	129	132	138	145
Na urine (mEq/L)							63		
pOsm (mOsm/Kg)		301	293		264	293	276	290	297
uOsm (mOsm/kg)		293		495	679		367	312	167
Diuresis				2500	4320				
Urine density					1015			1008	
Hb (g/dL)	10.9	10.9	9.1		11.6	10.4			10.1
Hematocrit (%)	31.1	32.7	27.5		32.5	28.8			29.3
NaCl 9 mg/mL (0.9%) 1000 mL		–	–	83 mL/h	42 mL/h	42 mL/h	42 mL/h	42 mL/h	42 mL/h
Hypertonic saline – NaCl 200 mg/mL (20%) diluted in fluid therapy	–	–	–	–	×3	×3	×6	×6	×4
Desmopressin (mg)		Nasal 0.05 mg/day			Stop				Started 0.03 mg/day
Fludrocortisone						0.1 mg tid			

*CSWS* cerebral salt wasting syndrome, *DI* diabetes insipidus, *Hb* hemoglobin, *Na* sodium, *NaCl* sodium chloride, *pOsm* plasma osmolality, *tid* three times a day, *uOsm* urine osmolality

On the sixth postoperative day he was transferred to our neurosurgery ward, and medicated with desmopressin 0.05/day, levothyroxine 75 μg, hydrocortisone 30 mg/day, and 1000 mL of isotonic fluid. He was started on levothyroxine because his blood tests after surgery revealed hypopituitarism: thyroid-stimulating hormone (TSH) 0.08 uUI/mL, Free T4 1.23 ng/dL (0.7–1.48), free testosterone 1.47 pg/mL (7.20–23), plasma cortisol 0.80 μg/dL, and adrenocorticotropic hormone (ACTH) < 1.0 ng/dL.

On the seventh postoperative day, he became confused and complained of headache. A cerebral computed tomography (CT) scan was performed with no significant changes. His blood tests showed that natremia dropped from 137 mEq/L to 128 mEq/l, with development of polyuria of 4320 mL/day, despite the maintenance of desmopressin dose. His hemoglobin and hematocrit rose from 9.1 g/L to 11.6 g/L and 27.5% to 32.5% (reference value 43–55), respectively. His thyroid function was normal, and he had taken the prescribed hydrocortisone dose. His plasma glucose level was 89 mg/L, blood urea nitrogen (BUN) 28 mg/dL, creatinine 0.51 mg/dL, potassium 3.7 mEq/L, and chloride 93 mEq/L. At 12 p.m. he initiated 150 mg of hydrocortisone infusion, but his Na level did not increase. Plasma and uOsm were 264 mOsm/kg and 679 mOsm/kg, respectively. At 4 p.m. hydrocortisone infusion was increased to 200 mg in 500 mL of sodium chloride (NaCl) 9 mg/mL (0.9%), and hypertonic saline replacements were started with infusion of hypertonic Na over 20 minutes. Desmopressin was removed from the prescription. Despite these medications his natremia dropped even more, to 124 mEq/L. The hydrocortisone infusion was replaced with intravenously administered hydrocortisone 50 mg four times a day, and another infusion of hypertonic Na was performed. In the evening his Na was 123 mEq/L and he was dehydrated, with reduced turgor, dried oral mucosa, persistent polyuria and vomiting, normal heart rate, and blood pressure of 96/58 mmHg. Hyponatremia was very resistant to treatment despite hypertonic saline replacements, without improvements of serum Na levels. We opted to start a continued infusion of three ampoules of 20% hypertonic saline diluted in 1000 mL of isotonic saline with an infusion rate of 42 mL/hour, and he was transferred to an intermediate care unit for proper surveillance. Given this clinical picture, the association of DI and CSWS was considered. On the ninth postoperative day, urine spot analysis showed that natriuresis was 63 mEq/L, even in the face of decreased serum Na of 132 mEq/L, representing another clue to the confirmation of the CSWS diagnosis.

On the eight postoperative day our patient’s Na was 129 mEq/L, and we started fludrocortisone 0.1 mg/three times a day because it is a known effective adjunct treatment of CSWS. His Na increased to 132 on the following day. As an adverse effect of fludrocortisone, he developed hypokalemia, which we controlled with potassium supplements.

Over the following days, his Na level was stabilized and desmopressin was restarted. His urine volume subsequently decreased to a normal diuresis. He was managed with intravenously administered fluids and hypertonic saline. The hypertonic saline dose was gradually decreased and switched to NaCl tablets.

He was discharged on postoperative day 27, medicated with fludrocortisone 0.1 mg/twice a day, orally administered NaCl 16 g/day, orally administered desmopressin 0.1 mg/twice a day, hydrocortisone 20 mg/day, levothyroxine 100 mg/day, and potassium chloride supplements. On follow-up as an out-patient, fludrocortisone and potassium were reduced and then discontinued. Two months later he was only taking hydrocortisone, desmopressin, and levothyroxine, and testosterone replacement was prescribed. The dose of desmopressin had to be increased to 0.5 mg/day to control diuresis, which indicated persistent DI in our patient.

Regarding the adenoma, the histological result was revealed to be a gonadotropinoma. He underwent another neurosurgery on February 4, 2016; he also underwent external radiotherapy with a total dose of 52.2 Gy in 29 fractions and with photon energy of 18 MV, according to the computerized dosimetry planning. He is now clinically well, with his hypopituitarism and permanent DI controlled.

## Discussion and conclusions

This case describes a patient with a rare combination of two diseases involving different electrolyte disturbances. DI typically occurs as early as several hours after brain surgery, usually within the first 24 to 48 hours after surgery, as was the case in this patient [13, 15]. Diagnosis is made based on clinical and biochemical results, which include sudden onset of polyuria, polydipsia, high serum Na, high pOsm, low uOsm, and low urine specific gravity. In cases of high urine output it is also important to rule out glycosuria and hyperglycemia, especially if the patient is on steroids, and to check if intraoperative fluid overload could have contributed to polyuria [1, 15]. However, in these cases, the low level of Na is an artifact in the presence of hyperglycemia and glycosuria, and typically natremia is normal or low and there is no increase in thirst with fluids overload [5]. This patient was on dexamethasone 4 mg/day, but his glycaemia was in the normal range and he was not suffering from fluid overload.

There are several risk factors for the development of DI, namely: being of young age; being male; and having larger sellar tumors (as this patient had), intraoperative cerebrospinal fluid fistula, Rathke’s cysts, or craniopharyngioma [5]. The treatment of this condition is relatively easy with desmopressin. However, this treatment should be monitored to assess whether the desmopressin dosage is correct to achieve a normal Na value and to prevent overdose and subsequent hyponatremia.

When the hyponatremia of this patient began he was on desmopressin, but he developed profound polyuria, which indicates that an excessive dose of desmopressin was not the cause. In cases of desmopressin deficiency, hypernatremia is seen with polyuria, as occurred on the first day after surgery with this patient.

One study has reported that, very often, the cause of hyponatremia is not clear [16]. This patient initially had moderately severe symptoms of hyponatremia, but then developed severe symptoms, with his hyponatremia worsening from 128 mEq/L, moderate hyponatremia, to 124 mEq/L, profound hyponatremia. There are many potential causes of hyponatremia after pituitary surgery: hypertonic fluid overload during surgery, SIADH by posterior lobe lesion, CSWS, secondary adrenal insufficiency, central hypothyroidism, desmopressin intoxication, hyperglycemia, and drugs. However, there are some clues that should be considered in order to make a diagnosis. Iatrogenic overload of hypotonic fluids and secondary adrenal insufficiency are more common in the first few days after surgery, while SIADH and CSWS occur typically 1 week after surgery or later. SIADH and hypothyroidism are associated with euvolemia, and CSWS is associated with hypovolemia [5, 12]. This patient was on 30 mg/day hydrocortisone and 75 μg/day levothyroxine with a normal thyroxine value, so it was unlikely that adrenal insufficiency and hypothyroidism were causing hyponatremia. Despite that, hydrocortisone was increased and Na continued to decrease and hypothyroidism very rarely causes hyponatremia, even though it is mentioned in many diagnostic algorithms [12].

CSWS usually occurs in the first week after subarachnoid hemorrhage, trauma, stroke, or brain surgery, and resolves spontaneously after 3 to 4 weeks. In rare cases it can be long-standing and can last for months, especially in the presence of CNS infection, cerebrospinal fluid obstruction, and tumor progression. A diagnosis of CSWS has to be considered in the presence of hyponatremia, an inappropriately elevated uOsm, high urine volume, urine Na concentration greater that 30 mEq/L, low serum uric acid concentration due to urate wasting in the urine, orthostatic hypotension, tachycardia, poor skin turgor, and low central venous pressure [12, 17]. It is not rare to see an increase in hematocrit and urea levels, which indicate dehydration. The most important criterion to differentiate CSWS from SIADH is extracellular fluid volume status, which is increased in SIADH and decreased in CSWS. Weight is increased or unchanged in SIADH, and decreased in CSWS; serum osmolality is decreased in SIADH and increased or normal in CSWS, serum protein levels are normal or high in CSWS, and hematocrit is low or normal in SIADH and increased in CSWS [7]. An appropriate examination is crucial for correct diagnosis, and it is important to remember that there are some laboratory findings that are seen in SIADH and sometimes the patient has milder hypovolemia, in which case it is challenging to distinguish this from SIADH [15, 17]. In the case of this patient this doubt did not exist, as he developed an obvious clinical condition of hypovolemia and dehydration. Unfortunately, we do not have information regarding the weight of our patient nor his water balance when he developed CSWS because he was on a neurosurgery ward at the time.

According to several recommendations or guidelines, in the presence of acute hyponatremia hypertonic saline solution should be administered, as we did [7, 12]. Moreover, the distinction between CSWS and SIADH is critically important since the two disorders are managed differently, with potential mortal effects if the incorrect strategy is administered. Treatment of CSWS involves water and salt supplementation since volume repletion will suppress the release of ADH, resulting in dilute urine and correction of hyponatremia. In the presence of SIADH, isotonic saline often worsens the hyponatremia as the salt is excreted and some of the water is retained, so the treatment must be fluid restriction. Mineralocorticoid administration, fludrocortisone 0.2–0.4 mg/day, is often indicated for the treatment of SIADH. This drug mediates a return to normal Na values via a reduction in natriuresis by increasing Na reabsorption in the renal proximal tubule [1, 15, 18]. Hypokalemia is a common adverse effect, but it can be managed by adding potassium supplements, as we did [15]. It is not known when the ideal time to start fludrocortisone is, but some authors suggest that it should be started after several days when the diagnosis is not clear, when management by replacement of salt/fluids is not sufficient, or when it is causing practical difficulties [18].

When CSWS coexists with DI, polyuria secondary to natriuresis should not be considered a sign of poorly controlled DI [1, 15]. In DI, hypernatremia is characteristic, whereas in CSWS natremia typically decreases and an increase of the desmopressin dose may worsen hyponatremia [15, 19]. We opted to stop desmopressin for 5 days, but some authors have reported keeping desmopressin therapeutic with careful surveillance of fluid and natremia status [13].

There are only a few reports described in the literature of this combination of diagnoses in the same patient, and this association is linked to very high mortality [8, 13, 14].

In a review of 16 children with DI plus CSWS, only three patients survived in a vegetative state. In another study of 54 children with DI, 16 developed CSWS and only two of these survived, also in a vegetative state. In these two reviews, the most common etiology was CNS infection, and none had pituitary surgery as the cause [8, 14].

The last published review regarding patients with this combination of diseases included 11 patients, mostly adults, with traumatic brain injuries caused by traffic accidents or falls. Traumatic subarachnoid hemorrhage was the major cause of this association, and only five patients had a good prognosis. The authors noted that the fact that serum Na levels might be normal in combined DI and CSWS can contribute to a late diagnosis. Incorrect diagnosis and treatment strategy are likely to be responsible for these terrible results [13].

None of the previous reviews included patients for whom transsphenoidal surgery was the cause of the combination of diseases, and we found no previous case reports of this combination after transsphenoidal surgery.

In conclusion, the management of fluid and electrolyte disorders after brain surgery is challenging. DI and CSWS can occur in the same patient after pituitary surgery, and clinicians should be aware of this possibility. This case report highlights the importance of regularly reviewing the patient’s clinical situation, hydration status, urine output, and Na balance. This combination of conditions should be kept in mind in the postoperative period of cranial tumors, because a prompt diagnosis and appropriate therapy are necessary for a good outcome.

This is the first described case report in the literature of CSWS and DI after pituitary adenoma surgery with an unusual good outcome.

## Abbreviations

ADH: Antidiuretic hormone
CNS: Central nervous system
CSWS: Cerebral salt wasting syndrome
DI: Diabetes insipidus
Na: Sodium
pOsm: Plasma osmolality
SIADH: Syndrome of inappropriate antidiuretic hormone secretion
uOsm: Urine osmolality
